# Evaluation of cerebral microcirculation in a mouse model of systemic inflammation

**DOI:** 10.1117/1.NPh.11.3.035003

**Published:** 2024-07-15

**Authors:** Cong Zhang, Mohammad Jamshidi, Patrick Delafontaine-Martel, Andreas A Linninger, Frédéric Lesage

**Affiliations:** a Polytechnique Montreal, Department of Electrical Engineering, Montreal, Quebec, Canada; b Montreal Heart Institute, Research center, Montreal, Quebec, Canada; c University of Illinois at Chicago, Department of Biomedical Engineering, Chicago, Illinois, United States; d University of Illinois at Chicago, Department of Neurosurgery, Chicago, Illinois, United States

**Keywords:** capillary stalling, systemic inflammation, cerebral microcirculation, mean transit time, optical imaging

## Abstract

**Significance:**

Perturbations in the microcirculatory system have been observed in neurological conditions, such as Alzheimer’s disease or systemic inflammation. However, changes occurring at the level of the capillary are difficult to translate to biomarkers that could be measured macroscopically.

**Aim:**

We aim to evaluate whether transit time changes reflect capillary stalling and to what degree.

**Approach:**

We employ a combined spectral optical coherence tomography (OCT) and fluorescence optical imaging (FOI) system to investigate the relation between capillary stalling and transit time in a mouse model of systemic inflammation induced by intraperitoneal injection of lipopolysaccharide. Angiograms are obtained using OCT, and fluorescence signal images are acquired by the FOI system upon intravenous injection of fluorescein isothiocyanate via a catheter inserted into the tail vein.

**Results:**

Our findings reveal that lipopolysaccharide (LPS) administration significantly increases both the percentage and duration of capillary stalling compared to mice receiving a 0.9% saline injection. Moreover, LPS-induced mice exhibit significantly prolonged arteriovenous transit time compared to control mice.

**Conclusions:**

These observations suggest that capillary stalling, induced by inflammation, modulates cerebral mean transit time, a measure that has translational potential.

## 1 Introduction

Brain microcirculation is the intricate network of blood vessels encompassing arterioles, capillaries, and venules that delivers oxygen and nutrition and removes waste products necessary for the proper functioning of brain tissue.^1^^,^^2^ It plays a critical role in maintaining homeostasis associated with metabolic requirements and oxygen distribution to the tissue in the brain. In particular, brain capillaries play a pivotal role in facilitating the exchange of essential nutrients and oxygen to support neurons. However, in the presence of neurological disorders, such as Alzheimer’s disease^3^ or stroke,^4^^–^^6^ this microcirculatory system can be disrupted due to capillary stalling.

Given that stalling has been shown to occur in only a small fraction of capillaries, it is challenging to quantify. Traditional two-photon microscopy imaging was previously used to measure capillary stalling events,^3^^,^^6^^,^^7^ but the method requires acquiring images of individual capillaries in a relatively narrow field of view. Furthermore, the slow acquisition speed makes it difficult to simultaneously visualize multiple capillaries inside a larger volume on a wider scale. The requirement of injecting contrast agents for the visualization of vascular structures may also alter the phenomenon at hand. This led to the application of optical coherence tomography (OCT),^8^^–^^10^ which can obtain volumetric angiograms at a high speed while preserving capillary-level resolution without exogenous contrast. It thus provides an efficient method for studying the mechanism of capillary stalling.

Systemic inflammation is a prevalent pathological condition that causes significant stress on the vascular system,^11^^–^^13^ which is a significant factor in the development of neurodegenerative disease.^14^^,^^15^ The increased number of white blood cells during systemic inflammation presents a deleterious effect for the microcirculation, leading to potential capillary blockage of individual segments or entire clusters. Systemic inflammation can be triggered by lipopolysaccharide (LPS) injections, which is a key component of the outer membrane of gram-negative bacteria.^12^^,^^16^ In this study, we applied *in vivo* optical imaging techniques to examine alterations in cerebral vascular blood flow dynamics within a model of LPS-induced systemic inflammation. The combined spectral domain OCT and fluorescent optical imaging (FOI) system was used to monitor the resting-state capillary perfusion and microcirculation in the somatosensory barrel cortex of anesthetized mice. We found that LPS administration significantly increased both the percentage and duration of capillary stalling compared to mice receiving a 0.9% saline injection. Furthermore, LPS-induced mice exhibit significantly prolonged arteriovenous transit time compared to control mice, suggesting that the stalled vessels in the network impede tracer transit. Numerical simulations in realistic mouse angiograms were performed to compare the experimental data to stalling simulations and support our observations.

## 2 Materials and Methods

### 2.1 Animal Preparation and Surgical Procedure

All experiments and animal preparation were approved by the Animal Research Ethics Committee of the Montreal Heart Institute and were conducted in compliance with the Canadian Council on Animal Care recommendations. Twelve-week-old male and female C57BL/6 mice (wild type, bodyweight of 23±0.6  g, n=22) were purchased from Charles-River Laboratories. Two out of the initial 22 mice were excluded from this study due to post-surgery cranial bleeding complications and tail catheter installation failure. The mice were divided into two groups: a control group, which received a placebo injection of 200  μL 0.9% saline, and an experimental group with a systemic inflammatory response. This inflammation was triggered by the intraperitoneal administration of LPS purified from *E. coli* strain O111:B4 (Sigma-Aldrich, St. Louis, United States) at a single dose of 2  mg/kg in 200  μL saline,^17^^,^^18^ administered prior to the surgical procedure. The surgical procedure involved the placement of a titanium head bar and the installation of a cranial window, following a methodology previously described.^19^^–^^22^ Anesthesia was induced and maintained with 2% isoflurane and 2  L/min oxygen throughout the approximately 90-min surgical process. The hair over the brain was removed, and the skin was sterilized with iodine and 70% alcohol. 200  μL of lidocaine was subcutaneously applied at the surgical site for local analgesia. To prevent brain movement during subsequent imaging sessions, a titanium bar was securely fixed to the rear of the skull bone with dental cement. A craniotomy window was carefully created over the left somatosensory cortex (A/P, −1.5  mm; M/L, −3  mm) using a microdrill, and a 3 mm coverslip was placed on the cortical surface and glued with dental cement. During this procedure, the dura matter was kept intact. Throughout the entirety of the surgical procedure, the heart rate was carefully maintained at approximately 400 beats per minute, and the temperature was monitored and maintained at 37°C using a feedback-controlled heating platform (Labeo Tech). The respiration rate was maintained between 80 and 130 breaths per minute.

### 2.2 Combined Spectral-Domain Optical Coherence Tomography and Fluorescent Optical Imaging

**Fig. 1:**
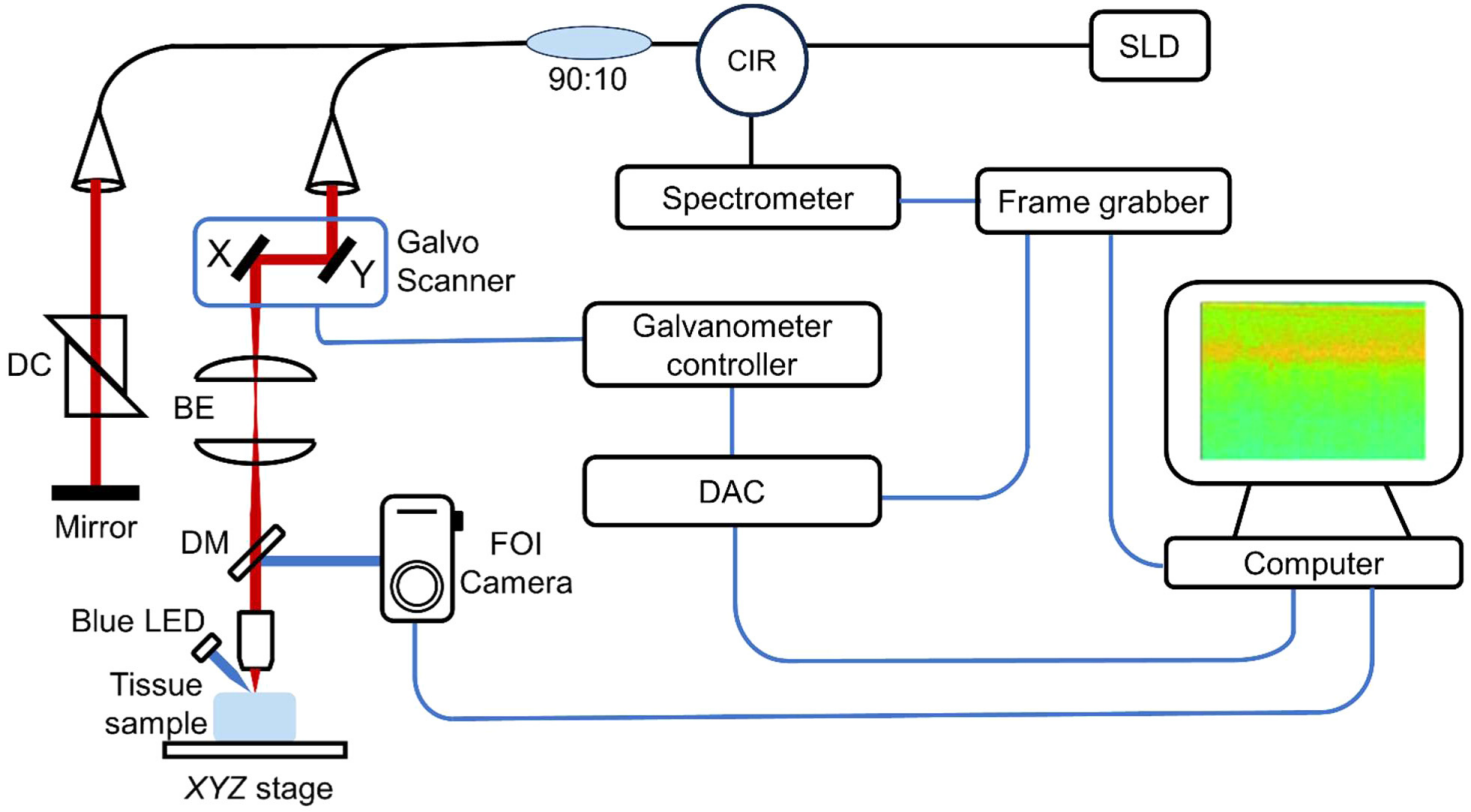
Schematic illustration of the combined spectral-domain OCT and FOI system. Within the OCT system, a SLD emitted infrared light at 1310 nm, which was then divided into the reference and sample arms through a fiber optic coupler. The light in the sample arm traversed a galvo scanner and a telescope and was focused onto the sample by an objective lens. The reflected light from the sample and the reference arm were collected by a spectrometer and digitized using a frame grabber. In the FOI system, the sample’s surface was illuminated by a blue LED, and the reflected light was directed to a camera through a dichroic mirror (DM). DC, dispersion compensator; 90:10, fiber optic coupler; DAC, data acquisition card; COL, collimator; and LED, light-emitting diode.

Data acquisition was performed using a custom-built system that combined spectral-domain OCT^23^ with FOI.^24^ A schematic representation of this integrated system is presented in Fig. 1. In this study, we utilized a broadband light source centered at 1310 nm, generated by a superluminescent diode (SLD) (LS2000C, Thorlabs) for the OCT system. The light was divided between the sample arm and the reference arm through a 90:10 fiber optic coupler (TW1300R2A2, Thorlabs). To focus the collimated light onto the tissue sample, we used a long working distance objective (10X, Mitutoyo, Japan) at the termination of the sample arm. Spectral interferograms were captured using a spectrometer (Cobra 1300, Wasatch Photonics) and digitized with a frame grabber (PCIe-1433, National Instruments). The axial resolution was 4.5  μm in biological tissues, with a lateral resolution of approximately 3.5  μm. The sample arm included a galvanometer scanner, telescope, and objective lens. The sample was affixed to an XYZ translation stage. This arrangement allowed for precise adjustment of the depth of the imaging focal point within the mouse cortex.

For FOI, a blue light emitting diode (LED) light source (CREE XPEBBL-L1, filtered at 472±30  nm) was utilized to excite FITC. Dichroic and emission filters (Thorlabs, DMLP 505R and Semrock FF01-496/LP-25) were placed before the camera (sCMOS, CS2100M-USB, Thorlabs) to permit the separation of the blue light and the emission signal from the sample. The camera was utilized to capture wide-field images and fluorescence signals emitted from the sample. A custom software interface using C++ was developed to operate both systems. This software allowed for the coordinated control and synchronization of the OCT and FOI components.

### 2.3 Imaging Protocol

**Fig. 2:**
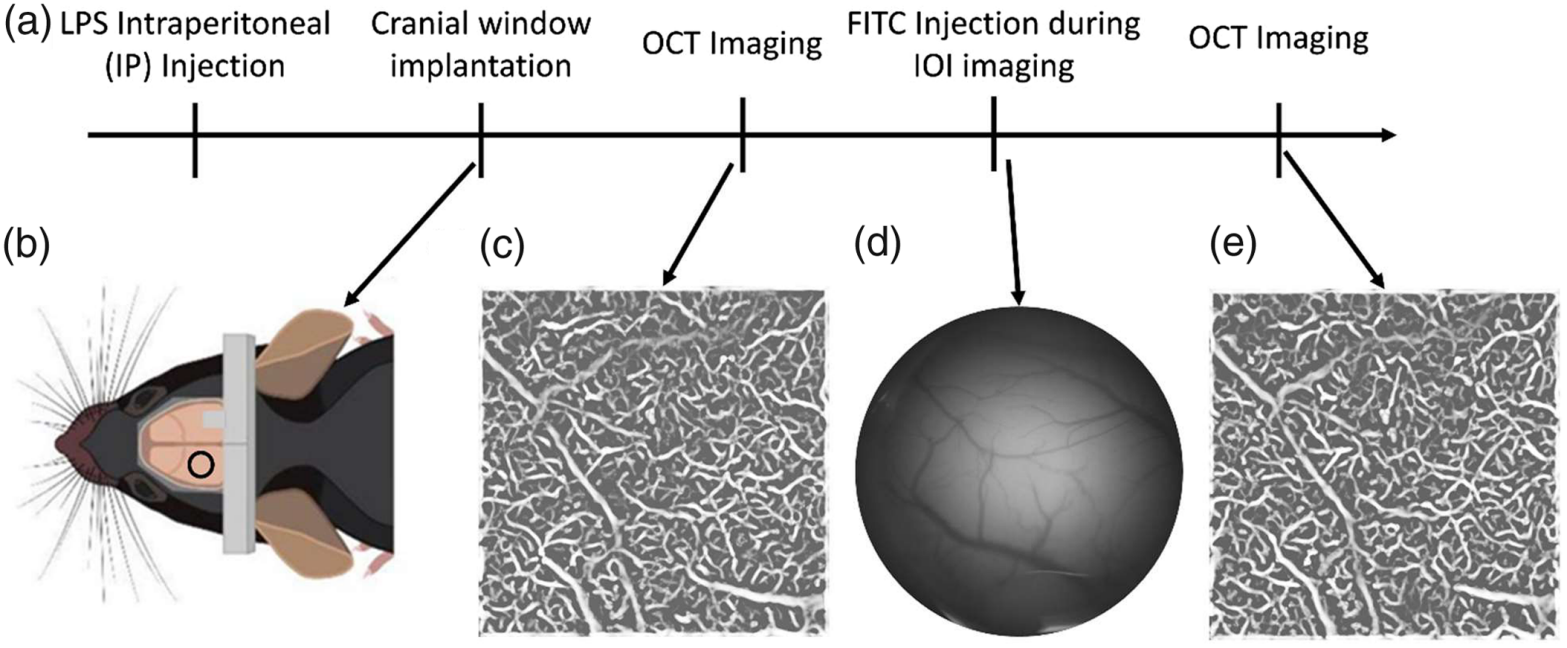
(a) Timeline of the experimental procedure. (b) Image of a mouse with the cranial window implanted. (c) and (e) Example of a filtered angiogram acquired with the OCT system. (d) Example image captured with the FOI system.

Following the craniotomy surgery, the mouse was subsequently positioned on the imaging system stage. A 30-gauge catheter needle was aseptically inserted into the tail vein, and a pump (Harvard Apparatus PHD 2000) was employed to facilitate controlled injections for transit time measures. During this procedure and imaging, isoflurane (1.5% isoflurane with oxygen flow rate 1  L/min) was supplied by a nosecone. The entire experimental protocol is illustrated in Fig. 2. During the initial phase of each experiment, OCT was utilized to acquire angiographic data from a designated region of interest (ROI) measuring $1.2\times 1.2\;mm^{2}$ under baseline conditions. The focal plane depth for OCT angiogram acquisition was approximately 150 to 350  μm below the cortical surface. Subsequently, a 40  μL volume of fluorescein isothiocyanate conjugated with dextran (FITC-dextran, 2MDa Sigma-Aldrich, St. Louis, United States), with a concentration of 17  mg/mL, was injected through the tail vein catheter with an injection rate set to 58  μL/s, i.e., the injection was executed within a rapid 0.7-second interval. Concurrently with this injection, fluorescence signal imaging was captured via FOI. Finally, the identical ROI of $1.2\times 1.2\;mm^{2}$ at the same depth was imaged once more using OCT after injection. For each OCT imaging session, a temporal series comprising 60 volumes was recorded, with each OCT-angiogram acquisition of 60 volumes lasting approximately 9 min.

## 3 Data Analysis

### 3.1 OCT

**Fig. 3:**
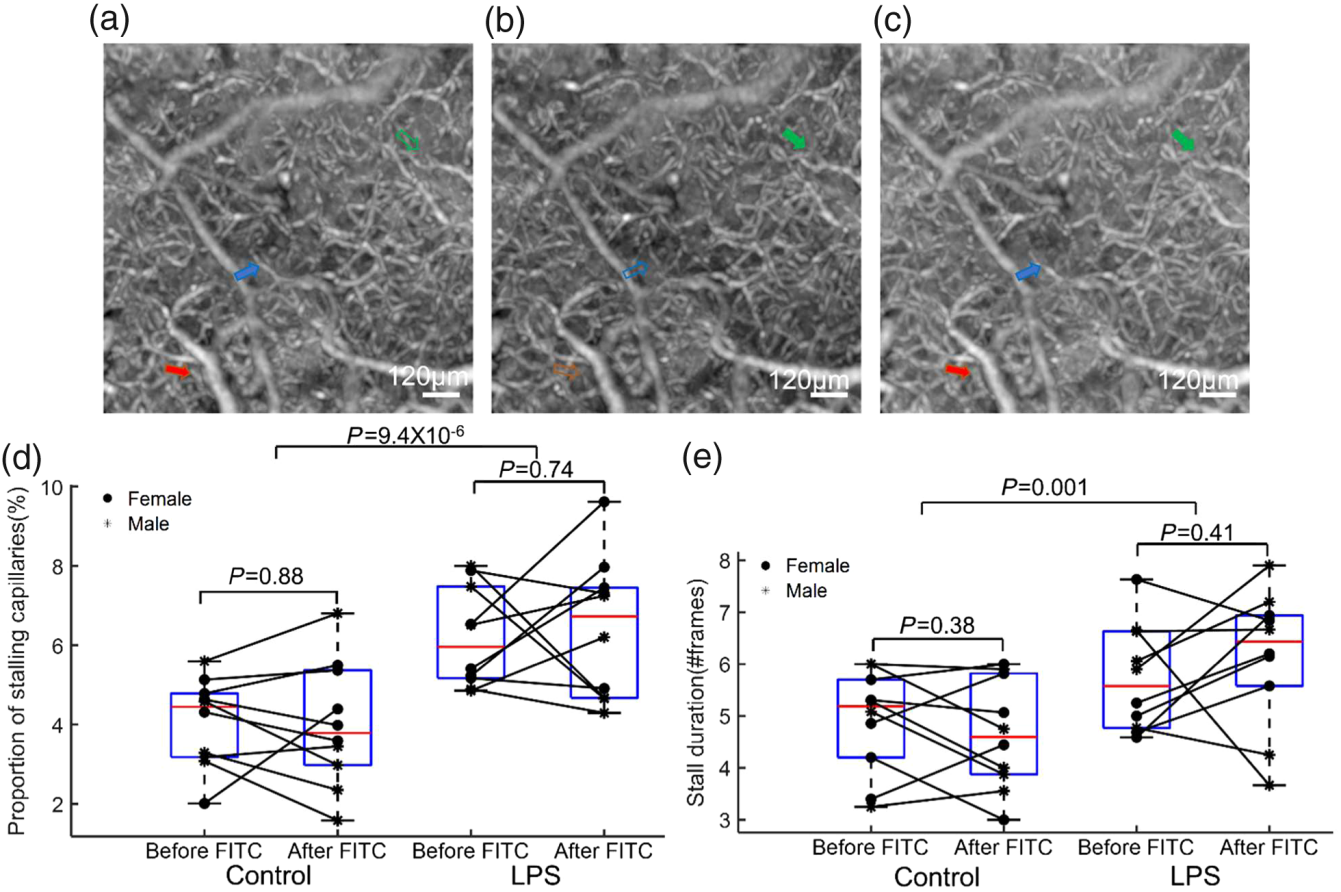
(a)–(c) Example of stalling capillary segments in an ROI. Each arrow indicates a capillary with flow, and the corresponding hollow arrow points to the same capillary where flow has ceased, resulting in stalling. (d) and (e) Quantification of capillary stalling events in the ROI. (d) Calculation of the proportion of capillaries exhibiting stalling events during a 9-min imaging session, capturing instances in which at least one stall event was recorded. (e) Measurement of the duration of individual stall events in no. of frames (∼9  s/frame). A cohort of 20 mice was employed to calculate these parameters, with one ROI per animal.

In the angiographic images, the presence of stagnant capillary segments was readily discernible due to a sudden decline in intensity, coinciding with the observable disappearance and reappearance of flowing red blood cells (RBCs) within these segments. However, the quantification of these instances of capillary stalling has traditionally entailed a labor-intensive and error-prone manual process. In the present study, we employed an open-source image processing analysis tool developed by S. Fruekilde.^8^^,^^10^ To outline the methodology briefly, the frames were initially transformed into 3D angiograms. Subsequently, 3D angiogram slabs were extracted and flattened *en face* using a maximum intensity projection technique along the Z-dimension, resulting in the generation of 60 flattened 512×512  pixels images (one per frame). Image filtering (vesselness filter) then effectively enhanced the contrast between the capillaries and the background. Following this, an automated segmentation and identification process was applied to delineate capillary structures, yielding approximately 200 to 300 segments per ROI within our dataset. Stall events were identified based on a decrease in image intensity, concomitant with the reappearance of flowing RBCs within these segments. To differentiate true stalling events from artificial signal fluctuations, a threshold was individually set for each capillary. The threshold level was determined manually for a single acquisition, and validated for visual accuracy, on separate acquisitions. Following validation, the process was automatized based on the optimal value set in these analyses. In this study, we established a minimum stall duration criterion of three frames, equivalent to approximately 27 s, given the inherent challenge of distinguishing “true” stalling events from noise. An example of stalling events is presented in Figs. 3(a)–3(c). The proportion of capillary stalling was calculated by the number of “true” stalling events divided by the total number capillary segments. Stall duration was quantified by the number of frames for “true” stalling events.

### 3.2 FOI

**Fig. 4:**
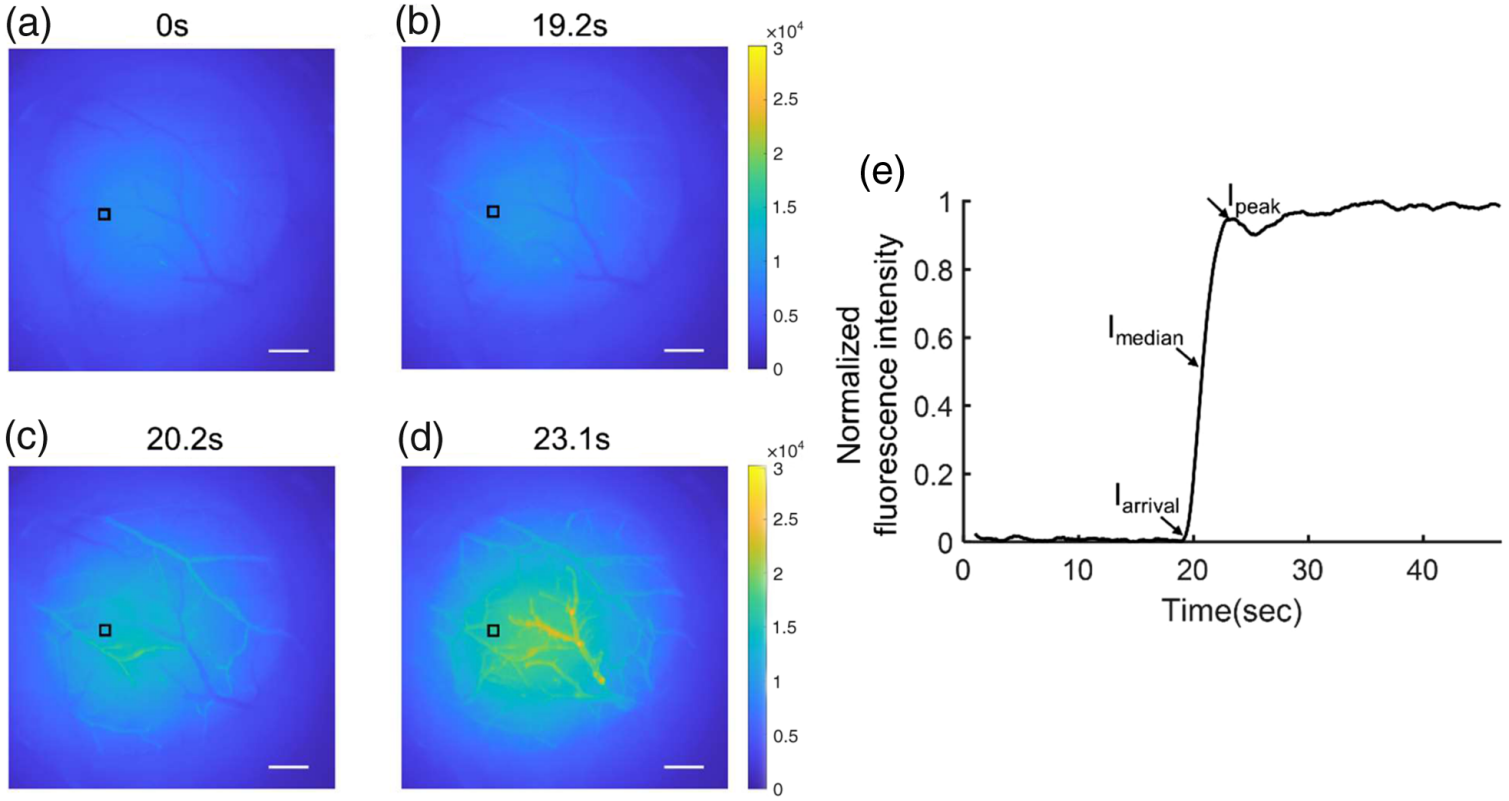
(a)–(d) Time-series acquisition of fluorescence imaging on a cranial window after intravenous bolus injection of FITC (scale bar: 0.3 mm). (e) Normalized fluorescence intensity dynamics in the indicated pixel (black square) post-FITC injection. $I_{\text{arrival}}$, $I_{\text{median}}$, and $I_{\text{peak}}$ were calculated based on alterations in the intensity.

During the FOI imaging, the cranial region was illuminated with an excitation wavelength of 472±20  nm. FITC-dextran was administered via a catheter in the tail vein. A time series of the FITC-dextran fluorescence excitation signal was acquired every 33.3 ms over approximately 45 s. Four examples of fluorescence signal changes observed during the injection are shown in Figs. 4(a)–4(d). For each mouse, ROIs on both arteries and veins were manually selected to compute the median value of the fluorescence intensity curve in each compartment. Subsequently, the arteriovenous mean transit time was calculated as the differences between the half-maximum rising time values of arteries and veins.^25^ The blood flow index was calculated by the ratio of difference in the fluorescence intensity and rise time as^26^^,^^27^

$$\mathrm{BFI}=\frac{I_{\text{peak}}-I_{\text{arrival}}}{T_{\text{peak}}-T_{\text{arrival}}},$$

where $I_{\text{peak}}$ is the initial peak of fluorescence intensity. $I_{\text{arrival}}$ is the first appearance of fluorescence intensity. $T_{\text{peak}}$ and $T_{\text{arrival}}$ are the time of peak and first appearance fluorescence intensity, respectively [Fig. 4(e)].

### 3.3 Statistical Analysis

All data are shown as the mean ± s.e.m for each group, with a sample size of n=5 mice per group. Comparison between the LPS-induced group and control group was conducted using two-sample t-test. A paired t-test was applied to analyze the differences between mice before and after the administration of the FITC-dextran injection.

## 4 Results

Data were collected from four distinct groups: control males, control females, LPS-treated males, and LPS-treated females, each comprising a sample size of n=5. The surgically implanted cranial windows facilitated the imaging of capillaries with the OCT system and enabled the clear detection of fluorescence changes in vessels during the administration of FITC-dextran via the tail injection. For each mouse, one ROI (1.2  mm×1.2  mm) was recorded two times (before and after the injection of FITC-dextran) by OCT imaging. A time series of FOI images from the whole cranial window were obtained during the injection of the FITC-dextran.

### 4.1 LPS Administration Leads to an Increase in the Percentage of Stalled Capillaries Whereas the Injection of FITC-Dextran did not Change the Stalling Properties

To study the effects of the LPS-induced inflammation on capillaries, OCT angiograms were acquired to examine the flow dynamics of capillaries. OCT images were obtained from the same ROI of the somatosensory cortex for ∼9  min before and after FITC-dextran injections. Capillary stalls were defined as the stoppage of blood flow in at least three subsequent imaging frames (∼27  s). Capillary stalling also occurred in controls;^3^^,^^5^^,^^28^^,^^29^ thus we quantified the proportion of stalled capillary segments that appeared in at least three frames [Fig. 3(d)] and the mean duration of individual stall events [Fig. 3(e)] in two groups. We also compared whether there were changes in capillary stalling caused by the intravenous FITC-dextran injection. A significant increase in all stall indices were found following LPS administration: the mean proportion of capillaries stalling ($p=9.4\times 10^{-6}$, P≪0.001) and the mean stall duration (p=0.001). However, no differences were observed before and after the injection of FITC-dextran in either the control (p=0.88) or LPS group (p=0.74) for the mean proportion of capillaries. Furthermore, there were no variations in stall duration before and after the FITC injection in either the control (p=0.38) or LPS group (p=0.41). The sex difference was also compared across all groups; no differences were revealed in this study.

### 4.2 Administration of LPS Induce a Longer Arteriovenous Transit Time and a Higher Blood Flow Index in the Pial Arteries and Veins

**Fig. 5:**
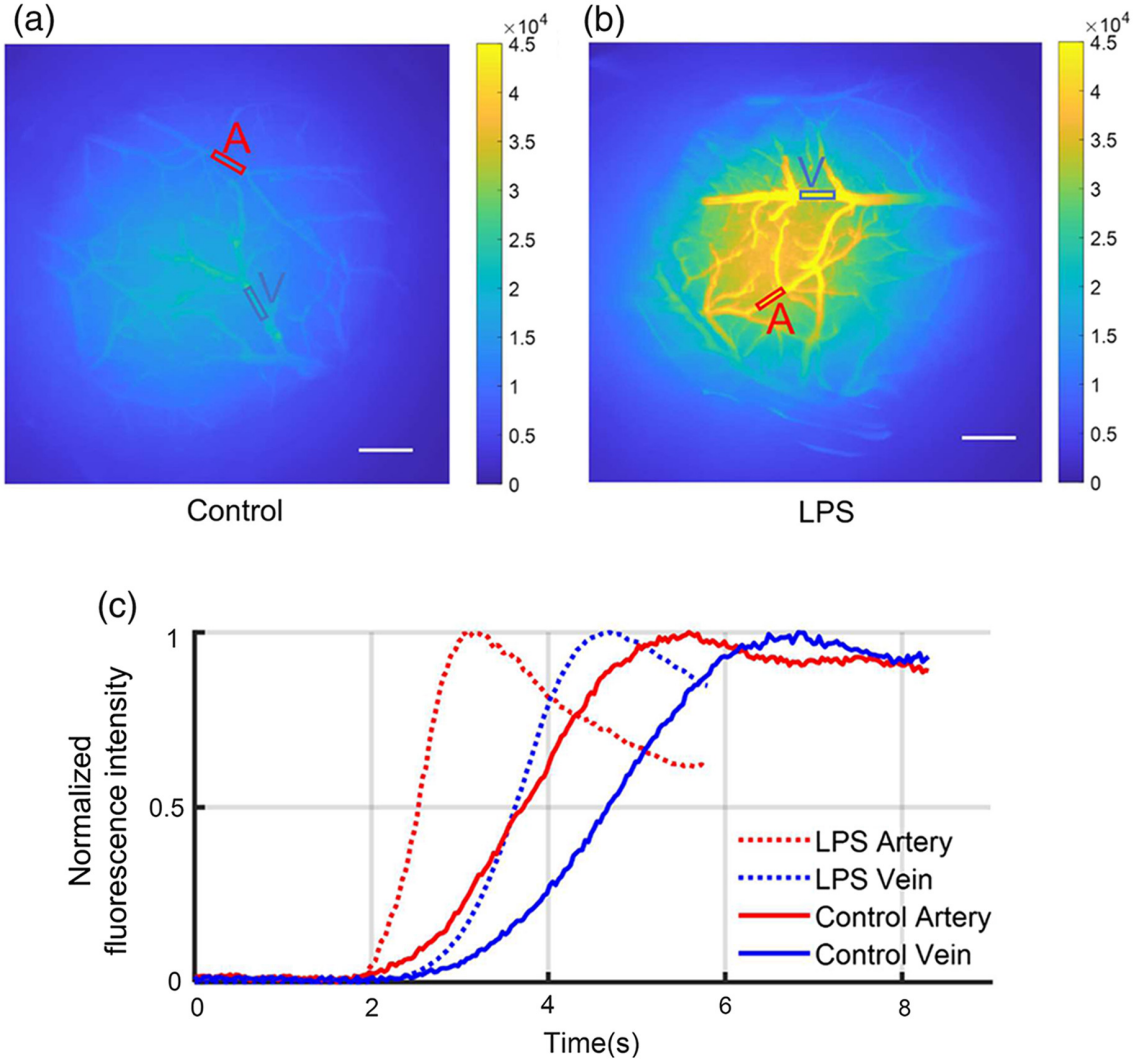
Example of fluorescence imaging during the FITC-dextran injection using the FOI system. (a), (b) Illustration of the ROIs corresponding to the artery and vein selected for analysis from the control group (a) and the LPS group (b) (scale bar: 0.3 mm). (c) Normalized fluorescence intensity alterations within the ROIs depicted in panels (a) and (b). Notably, the LPS group exhibits a greater arteriovenous transit time.

**Fig. 6:**
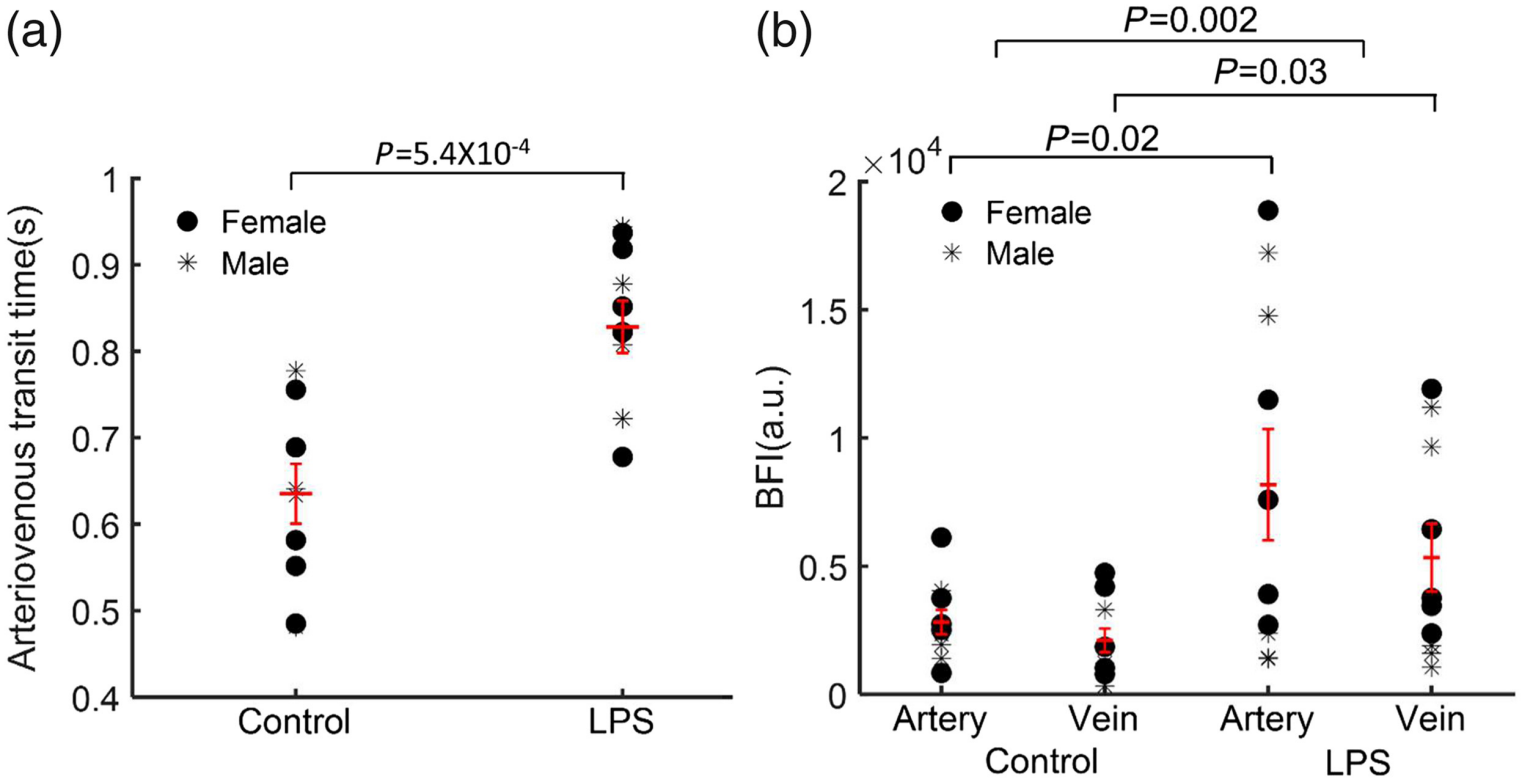
(a) Quantification of the arteriovenous mean transit time. LPS group exhibits a significant longer arteriovenous transit time compared to the control group. (b) Analysis of the blood flow index (BFI) for both arteries and veins in the control LPS groups. The LPS group shows higher BFI values in both arteries and veins compared to the control group.

Time-resolved fluorescence signal recordings could be used to distinguish arteries and veins because the signal first arrives in the arterial system and subsequently emerges in the pial venous network [Figs. 4(a)–4(d)]. Figures 5(a) and 5(b) illustrate examples of the selected ROIs (artery and vein) in both the control and LPS groups, and Fig. 5(c) displays the normalized fluorescence intensity in these regions. Although the absolute arrival time could not be compared due to experimental variability, we could quantify the arteriovenous transit time for all mice, as illustrated in Fig. 6(a). Remarkably, the administration of LPS elicited a substantial augmentation in arteriovenous transit time compared to the control group ($p=5.4\times 10^{-4}$, p≪0.001).

To elucidate the basis for the observed augmented arteriovenous mean transit time in response to LPS administration, the blood flow index was computed for both arteries and veins in all mice [Fig. 6(b)]. The results revealed increased variability and a heightened blood flow index within the arteries of the LPS-administrated group as opposed to the control group (p=0.02). Furthermore, the venous system exhibited analogous findings to the arterial counterparts (p=0.03) across both experimental conditions.

**Fig. 7:**
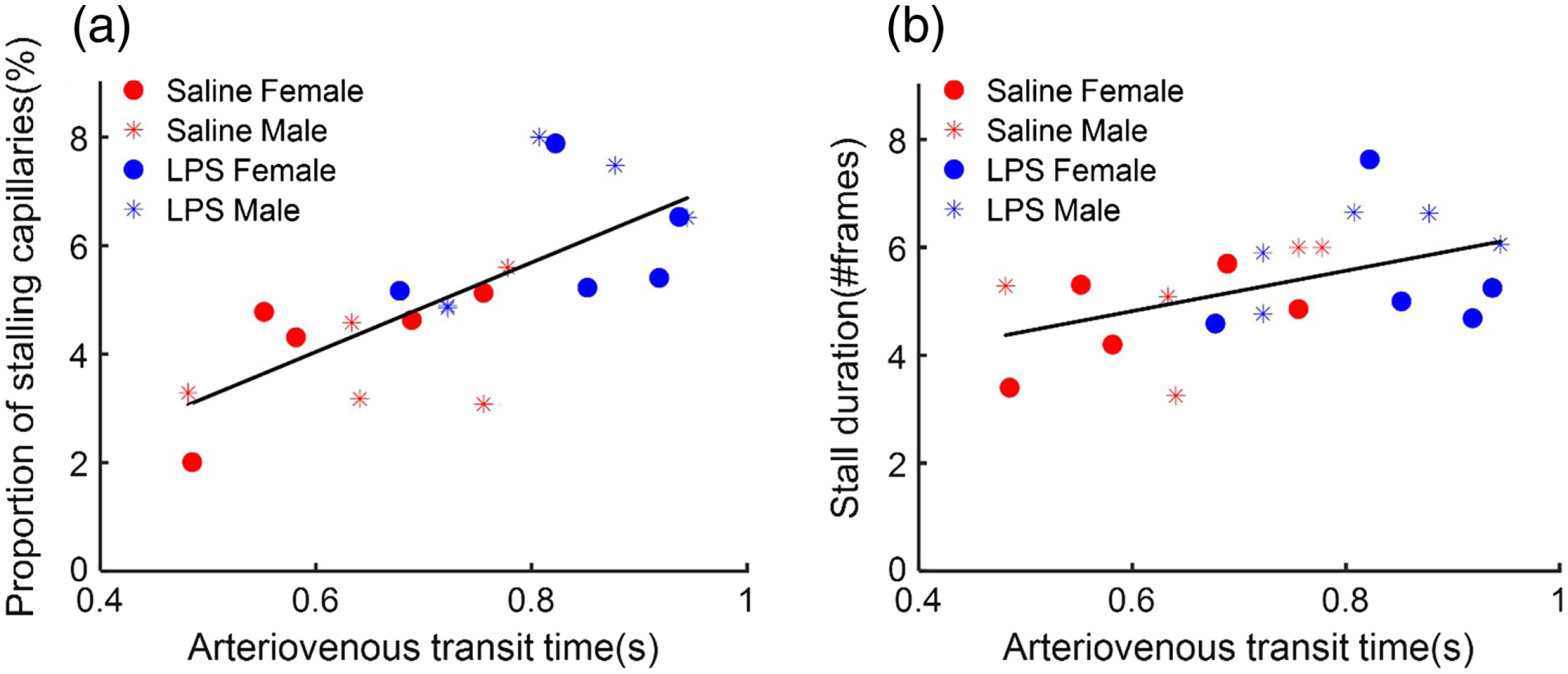
Scatter plotting illustrating the relationship between the capillary stalling and the arteriovenous transit time from arteries to veins. (a) Correlation between the arteriovenous transit time from arteries to veins and the proportion of stalling capillaries for all mice. The linear fit is y=8.24x−0.90, $R^{2}=0.53$, $P=2.7\times 10^{-4}$. (b) Correlation between the arteriovenous transit time from arteries to veins and the duration of stall events. The linear fit is y=3.75x+2.57, $R^{2}=0.25$, P=0.02. These parameters were analyzed across 20 mice, comprising 10 control mice (5 male, 5 female) and 10 LPS-induced mice (5 male, 5 female).

To validate whether the prolonged microcirculation time from arteries to veins observed in the LPS administration group is due to the capillaries stalling events, we characterized the relationship between the arteriovenous mean transit time and the occurrence of capillaries stalling. By plotting the arteriovenous mean transit time against the proportion of the stalling capillaries for all animals, a notable positive trend was reveled [Fig. 7(a), y=8.24x−0.90, $R^{2}=0.53$, $P=2.7\times 10^{-4}$]. This trend suggests that, as the proportion of capillaries experiencing stalling events increases, the corresponding microcirculation time from arteries to veins in the brain extends. Moreover, we performed the correlation between the arteriovenous transit time and the stall duration, and a positive relationship was also obtained [Fig. 7(b), y=3.75x+2.57, $R^{2}=0.25$, P=0.02]. This finding indicates that, with a longer stall duration, the microcirculation necessitates more time to traverse from arteries to vein.

### 4.3 Simulation of Stalling Events Using a Realistic Mouse Brain Supports the Increase in Transit Time

**Fig. 8:**
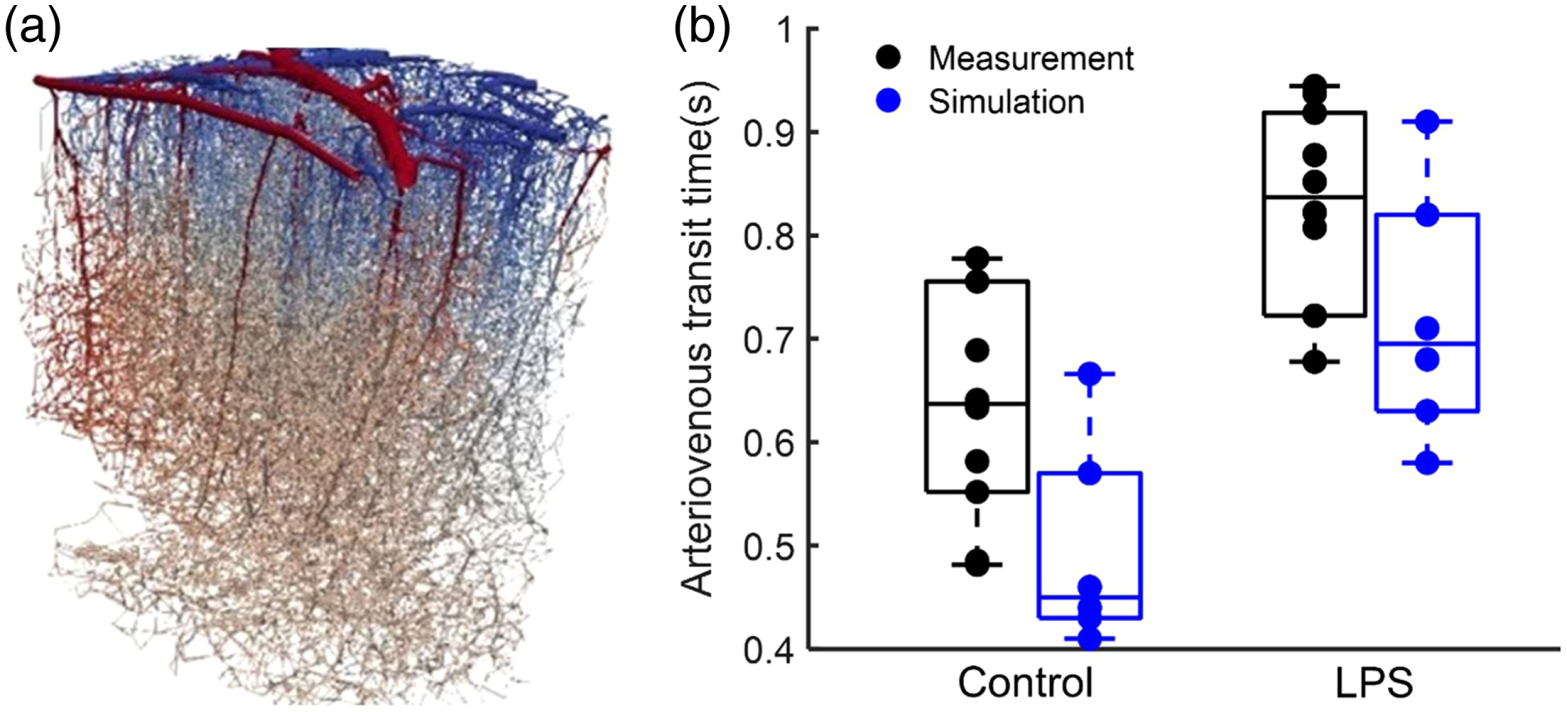
(a) Representation of the simulated network and (b) measurement and simulation of the time needed for blood to move through the arteriovenous networks. The arteriovenous transit time was assessed both before (control) and after simulating various stalling proportions. Results indicated a significantly longer average arteriovenous transit time in the LPS group when compared to the control group. In the simulation, each dot represents a stalling scenario: 0%, 0.5%, 1%, 2%, 2.5%, and 3% to model the control condition and 4%, 6%, 7%, 8%, 9%, and 10% to model the LPS condition.

Finally, to better understand the mechanistic origins of the observations, we exploited a recent work^30^ to simulate capillary blockage and its effect of transit time. Details of the simulation framework are described in Refs. 31 and 32. A synthetic microvascular network reproducing cortical network properties from the literature was used for simulations. Six vascular measurement points were set on the arterial and on the venous side respectively for spatial sampling of the transit response. To simulate stalling, and to model experiments, capillaries were blocked randomly in the network with stalling value proportions of 0%, 1%, and 3% to model the control condition and 4%, 8%, and 10% to model the LPS condition. Flow simulations tracking the tracer enabled the evaluation of transit between each artery and vein measurement point, leading to estimates for comparison. The arteriovenous transit time for both simulations and experiments is provided in Fig. 8, with the simulation outcomes indicating an extension in arterial to venous transit time due to stalls.

## 5 Discussion

In this study, we investigated the impact of systemic inflammation induced by the intraperitoneal injection of LPS on the cerebral vasculature and microcirculation in mice. Utilizing the combined spectra-domain OCT and fluorescence optical imaging (FOI) system, we studied the capillary stalling and cerebral arteriovenous mean transit time in the brain in response to the systemic inflammation. Our findings revealed a significant increase in both the proportion and duration of stalled flow events in response to LPS-induced inflammation. Concurrently, the blood flow index in arteries and veins, as well as the arteriovenous transit time, exhibited a notable increase in LPS group compared to the control group. These results support the conclusion that capillary stalling induced by systemic inflammation modulates the cerebral microcirculation.

### 5.1 Systemic Inflammation Induced Capillary Stalling

Our findings revealed a notable increase in the proportion of capillaries stalling and the blockage duration in response to the LPS injection. The increased prevalence of capillary stalls observed in the LPS-induced group could be explained by the upregulation of the circulating cytokines, followed by an increase in the number of lymphocytes, neutrophils, and monocytes in the bloodstream, which could present a challenge in the microcirculation.^33^^,^^34^

We observed that the proportion of stalling events obtained from the control group were slightly higher than those reported in a previous study that employed similar methods.^8^ We attribute this discrepancy to differences in our imaging recording conditions, specifically the use of anesthesia with isoflurane necessitated by the presence of a catheter in the tail vein for FITC injections. It is worth noting that isoflurane can induce a reduction in blood pressure,^35^ potentially enhancing capillary stalling.^9^ Moreover, the observed lower levels of capillary stalling in the LPS-injected group compared with a previous study^8^ could be due to the potentially lower dosage of LPS administered and the measure time points investigated. Additionally, we conducted experiments injecting FITC-dextran and found that it did not significantly alter the characteristic pattern of capillary stalling. This finding supports the broader application of FITC-dextran in studying blood flow dynamics with two-photon microscopy.^20^^,^^36^^,^^37^

### 5.2 Systemic Inflammation Delayed the Transit Through Cerebral Microcirculation

The assessment of arteriovenous mean transit time using FOI with bolus tracking enables the quantification of the plasma flow dynamics throughout the entire brain, reaching a depth of ∼1200  μm.^38^ Our results from FOI indicated an increase of the blood flow index in arteries and veins on the cortical surface in mice with systemic inflammation, which is consistent with previous observations of vasodilation after LPS injection.^8^ Moreover, an increase of arteriovenous transit time was shown in mice with systemic inflammation. This is consistent with previous observations of transit time increase from arterioles to venules in mice modeling Alzheimer’s disease,^39^ in which increased capillary stalling had already been observed.^3^ Inspired by previous studies of cerebral blood flow,^7^^,^^8^^,^^36^^,^^40^ we hypothesized that capillary stalling could be the primary cause of the longer transit in LPS-induced mice.

To further this hypothesis, we performed explicit simulations exploiting realistic angiographic digital twins and showed that, over multiple simulations, increased stalling leads to higher transit time and higher variability in transit time at higher stalls. These computational results support the link between transit time and stalling and potentially open new avenues to investigate macroscopic biomarkers of stalling.

Compared with traditional techniques utilized for studying of capillary stalling, including two-photon microscopy^36^ and OCT,^8^^,^^10^ it is noteworthy that the application of fluorescent optical imaging could represents a simpler approach in the investigation of capillary stalling. It offers a direct, expeditious, and comprehensive means of gaining insights into the mechanisms of micro cerebral circulation, and improving and specializing the simulations done here will potentially yield a validated biomarker that can help investigate this elusive phenomenon.

### 5.3 Limitations

LPS injections can also modify blood pressure, which could also modulate values of transit measures to the interpretation of the transit results. We performed simulations modifying blood pressure, and indeed, transit time was reduced, but to a lesser degree than by stalling. Future work investigating this aspect experimentally and assessing not only transit time but heterogeneity could help clarify the role of blood pressure on these biomarkers. Recordings were performed under isoflurane anesthesia, an agent that causes dilation of vessel diameters. This vessel dilation induced by isoflurane may not impact the primary conclusions of our study, i.e., comparing relative changes between the control and LPS-induced groups, both of which were subjected to the same anesthetic condition. Nonetheless, conducting a similar study in awake animals would be valuable and could warrant further exploration. Another limitation is the application of a minimal capillary stalling time for identifying the “true stalls” in OCT analysis, potentially leading to the oversight of capillary stalling instances. This limitation could be addressed by the utilization of a higher-temporal resolution optical imaging system, enhancing the accuracy of stall quantification. Finally, simulations confirmed the increase in transit time with increased stalling, but the absolute values are dependent on the angioarchitecture variations; this will be the subject of investigations in future works.

## 6 Conclusion

In this study, a combined spectral OCT and intrinsic signal optical imaging system was applied to investigate the capillary stalling and arteriovenous transit time of cerebral blood vessels in a mouse model of systemic inflammation induced by the LPS. A significant increase in both proportion and duration of capillary stalling was found in the LPS-induced group compared to the control group. In addition, LPS-induced mice exhibited a prolonged arteriovenous transit time compared to mice receiving a 0.9% saline injection. These outcomes suggest that capillary stalling modulates the arteriovenous transit time of cerebral vessels, which supplies a measure that has translational potential.
